# Beta-lactamase determinants and molecular typing of carbapenem-resistant classic and hypervirulent *Klebsiella pneumoniae* clinical isolates from southwest of Iran

**DOI:** 10.3389/fmicb.2022.1029686

**Published:** 2022-11-03

**Authors:** Morteza Saki, Mansour Amin, Mohammad Savari, Mohammad Hashemzadeh, Seyed Saeid Seyedian

**Affiliations:** ^1^Infectious and Tropical Diseases Research Center, Health Research Institute, Ahvaz Jundishapur University of Medical Sciences, Ahvaz, Iran; ^2^Department of Microbiology, Faculty of Medicine, Ahvaz Jundishapur University of Medical Sciences, Ahvaz, Iran; ^3^Alimentary Tract Research Center, Ahvaz Jundishapur University of Medical Sciences, Ahvaz, Iran

**Keywords:** beta-lactamase, carbapenem-resistant, ESBLs, hypervirulent *Klebsiella pneumoniae*, Iran

## Abstract

This study investigated the molecular epidemiology of carbapenem-resistant classic *Klebsiella pneumoniae* (CR-cKp) and carbapenem-resistant hypervirulent *Klebsiella pneumoniae* (CR-hvKp) isolates in southwestern Iran. From 2019 to 2021, 136 (88.9%) cKp and 17 (11.1%) hvKp isolates were identified using biochemical tests and polymerase chain reaction (PCR). Antibiotic resistance, beta-lactamases, and clonal relatedness of carbapenem-resistant isolates were investigated using disk diffusion, PCR, and enterobacterial repetitive intergenic consensus polymerase chain reaction (ERIC-PCR), respectively. The different markers of hvKp isolates were as follows: string test (35.3%, *n* = 6/17), *magA* (11.8%, *n* = 2/17), *rmpA* (11.8%, *n* = 2/17), *rmpA2* (52.9%, *n* = 9/17), *iucA* (52.9%, *n* = 9/17), and *peg344* (35.3%, *n* = 6/17). Also, 55.1% (*n* = 75/136) of cKp and 47.1% (*n* = 8/17) of hvKp isolates were CR-cKp and CR-hvKp, respectively. All CR-hvKp (100.0%, *n* = 8) isolates were MDR. Colistin, tetracycline, and tigecycline were the most effective antibiotics. The occurrence of beta-lactamase genes in 75 CR-cKp and 8 CR-hvKp isolates was as follows: *bla*_NDM_ (41.3, 25.0%), *bla*_IMP_ (4.0, 0.0%), *bla*_VIM_ (8.0, 0.0%), *bla*_GES_ (14.7, 25.0%), *bla*_OXA–48–like_ (20.0, 0.0%), *bla*_CTX–M_ (26.7, 12.5%), *bla*_SHV_ (24.0, 12.5%), *bla*_TEM_ (10.7, 0.0%), *bla*_FOX_ (6.7, 0.0%), *bla*_DHA_ (6.7, 0.0%), *bla*_CMY_ (5.3, 0.0%), *bla*_LAT_ (12.0, 0.0%), and *bla*_ACT_ (8.0, 0.0%). ERIC-PCR showed a high diversity among isolates. In this study, the occurrence of MDR CR-hvKp isolates harboring *bla*_NDM_ and *bla*_GES_ was detected for the first time in southwestern Iran. To prevent the spread of CR-hvKp and reduce selection pressure, long-term surveillance and more effective treatment strategies should be implemented.

## Introduction

*Klebsiella pneumoniae* is one of the most important Gram-negative bacteria (GNB) causing a variety of community-acquired and nosocomial infections. It is estimated that about one-third of all Gram-negative infections are caused by this bacterium (Maleki et al., 2018; Effah et al., 2020). Hypervirulent *K. pneumoniae* (hvKp) characterized by hypermucoviscosity was first reported from a liver abscess with extrahepatic complications, including endophthalmitis, in Taiwan in 1986. HvKp has been implicated in community-associated infections in healthy people with the most common cases of pyogenic liver abscesses in Asia (Serban et al., 2021; Zhu et al., 2021).

Some phenotypic and genotypic distinctive properties and determining factors differentiate classic *Klebsiella pneumoniae* (cKp) strains from hvKp. These include the string test, the regulator of mucoid phenotype A and A2 (*rmpA* and *rmpA2*), an aerobactin siderophore (*iucA*), the *peg-344* gene, and the *mucoviscosity-associated gene A* (*magA*) (Liao et al., 2020; Serban et al., 2021; Zhu et al., 2021). The string test that is based on the hypermucoviscosity of colonies, has been used to detect hvKp with high accuracy. However, hvKp strains without hypermucoviscosity and cKp strains with mucoviscosity were also found (Liao et al., 2020; Serban et al., 2021; Zhu et al., 2021). Therefore, the detection of hvKp is currently based on both phenotypic and genotypic characteristics.

Resistance to carbapenem antibiotics is of clinical importance because these drugs represent one of the last reserves in the treatment of infections caused by numerous multidrug-resistant (MDR) GNB, including *K. pneumoniae*. A recent systematic review and meta-analysis from Iran found a prevalence rate of 0.004–58% for carbapenem-resistant *K. pneumoniae* (CR-Kp) in different regions of the country (Vaez et al., 2019). According to this meta-analysis, CR-Kp is alarmingly common in the majority of Iranian hospitals (Vaez et al., 2019). However, there are limited data on the prevalence of CR-hvKp strains and associated mechanisms in Middle Eastern countries, including Iran.

A number of factors contribute to the difficulties in managing infections caused by CR-Kp, including simultaneous resistance to multiple antibiotic classes, poor patient clinical outcomes, and prolonged hospitalizations (Vaez et al., 2019). Resistance to beta-lactam antibiotics, including carbapenems, is mediated by a network of different mechanisms and genes (Dalmolin et al., 2017; Codjoe and Donkor, 2018; Shiri et al., 2020). The major mechanisms for carbapenem resistance in *K. pneumoniae* include the production of various beta-lactamases capable of hydrolyzing carbapenems as well as the reduced membrane permeability that occurs via loss or downregulation expression of outer membrane porins (OMPs) (Dalmolin et al., 2017; Codjoe and Donkor, 2018). CR-Kp infections are currently treated with limited therapeutic options including polymyxins, tigecycline, and ceftazidime/avibactam (Chen et al., 2021).

Although nearly 4900 different beta-lactamases have been identified so far, carbapenemases including *Klebsiella pneumoniae* carbapenemase (KPC), oxacillinases (OXA)-type enzymes such as OXA-48-like carbapenemases, Guiana extended-spectrum beta-lactamase (GES), imipenem-hydrolyzing beta-lactamase (IMI) and metallo-beta-lactamases (MBLs) including Verona integrin-encoded MBL (VIM), New Delhi MBL (NDM), and imipenemase (IMP) play a major role in the development of carbapenem resistance in *Enterobacteriaceae* (Dalmolin et al., 2017; De Angelis et al., 2020; Chen et al., 2021). Also, the overexpression of extended-spectrum beta-lactamases (ESBLs) and AmpC β-lactamases with loss of the OmpK35 and Ompk36 porins contribute to the carbapenem resistance in *K. pneumoniae* (Hamzaoui et al., 2018). Several ESBL genes are found on plasmids and are often derived from mutations in the TEM (Temoneira) and SHV (Sulphydryl variable) genes. Other frequent ESBLs include CTX-M (cefotaximase-Munchen), PER (*Pseudomonas* extended resistance), and VEB (Vietnamese extended-spectrum beta-lactamases) enzymes (De Angelis et al., 2020; Akpaka et al., 2021). Moreover, ESBL enzymes such as CTX-M-33 with carbapenemase activity have recently been identified (De Angelis et al., 2020).

In general, ESBL, AmpC, carbapenemase, and MBL enzymes are associated with MDR phenomenon, which means that less antibiotic options are available to treat infections. Therefore, it is critical to understand the epidemiology of these determinants. Since rare comprehensive studies have been conducted on the prevalence of hvKp isolates, especially CR-hvKp, their antibiotic resistance patterns, beta-lactamase resistance genes, and clonal relatedness in southwestern Iran, the present study aimed to shed light on these issues.

## Materials and methods

### Study design and area

In this three-year cross-sectional study (2019–2021), non-repetitive clinical isolates of *K. pneumoniae* were collected from various clinical specimens of patients who were referred to the four main teaching hospitals (Imam Khomeini, Golestan, Taleghani, and Razi) affiliated to the Ahvaz Jundishapur University of Medical Sciences, Ahvaz, Iran. Ahvaz is the capital of Khuzestan province which located at 31°20’ north latitude and 48°40’ east longitude, and ranks the second-largest city (with an area of about 215 km^2^) after Tehran, the capital of Iran (Alizadeh and Sharifi, 2020). The aforesaid hospitals receive countless patients annually from neighboring provinces in southwestern Iran, including Bushehr, Hormozgan, Ilam, and Kohgiluyeh and Boyer-Ahmad.

### Bacterial isolation and identification

All collected specimens were cultured aseptically on 5% sheep blood agar and MacConkey agar plates (Condalab, Madrid, Spain) and incubated aerobically for 18–24 h at 37°C. Primary identification of *K. pneumoniae* isolates was performed using a series of standard biochemical tests including Gram stain, triple sugar iron (TSI) agar, sulfur-indole-motility (SIM) agar, lysine iron agar (LIA), ornithine decarboxylase, citrate utilization, and urea hydrolysis (Mahon et al., 2018). All media were purchased from Merck Co., Darmstadt, Germany. *K. pneumoniae* ATCC^®^ 13883™ was used as control strain.

### Molecular confirmation of *Klebsiella pneumoniae* isolates

The presumptive *K. pneumoniae* isolates were confirmed by polymerase chain reaction (PCR) using specific primers for the 16S–23S internal transcribed spacer (*16S–23S ITS*) gene (130 bp), as previously described (Table 1; Abdel-Rhman, 2020). To extract genomic DNA, bacterial colonies were boiled in sterile distilled water for 10 min. After centrifugation at 14,000 rpm for 15 min, the supernatants were used as DNA template for PCR assay (Liao et al., 2020). All PCR reactions were performed in a final volume of 25 μl and consisted of 12.5 μl of master mix (Ampliqon, Denmark), 0.5 μl of each forward and reverse primer (10 pM), 1 μl of the DNA template, and 10.5 μl of nuclease-free water. PCR reactions were performed in a thermocycler (Biorad S1000 Thermal Cycler, USA) with the following program: initial denaturation at 95°C for 5 min, 35 cycles of denaturation at 95°C for 30 s, annealing (Table 1) for 30 s, and extension at 72°C for 45 s, followed by a final extension at 72°C for 5 min. *K. pneumoniae* ATCC^®^ 13883™ and nuclease-free water were used as positive and negative controls, respectively. Electrophoresis was performed on a 2% agarose gel stained with Safe Stain (Sinaclon Co., Tehran, Iran) to determine the size of PCR products compared to a 100 bp DNA ladder. The agarose gel was scanned using a UV light transilluminator (Protein Simple, San Jose, CA, USA). Confirmed isolates were stocked in tryptic soy broth (TSB) (Condalab, Madrid, Spain) containing 20% glycerol and stored at –80°C for ongoing analysis.

**TABLE 1:** The sequence of primers used in the polymerase chain reaction assay.

Genes/primers name	Sequences (5′–3′)	Annealing (°C)	Product size (bp)	References
*bla*_CTX–M_	F-CGTGCTGATGAGCGCTTTGC R-AGATCACCGCGATATCGTTG	59	568	Maleki et al., 2018
*16S–23S ITS*	F-ATTTGAAGAGGTTGCAAACGAT R-TTCACTCTGAAGTTTTCTTGTGTTC	58	130	Abdel-Rhman, 2020
*bla*_KPC_	F-CATTCAAGGGCTTTCTTGCTGC R-ACGACGGCATAGTCATTTGC	56	538	Abdel-Rhman, 2020
*bla*_OXA–48–like_	F-GCTTGATCGCCCTCGATT R-GATTTGCTCCGTGGCCGAAA	56	281	Abdel-Rhman, 2020
*bla*_NDM_	F-GGTTTGGCGATCTGGTTTTC R-CGGAATGGCTCATCACGATC	54	621	Abdel-Rhman, 2020
*bla*_VIM_	F-GATGGTGTTTGGTCGCATA R-CGAATGCGCAGCACCAG	55	390	Abdel-Rhman, 2020
ERIC	ERIC 1-ATGTAAGCTCCTGGGGATTCAC ERIC 2-AAGTAAGTGACTGGGGTGAGCG	49	Variable	Abdel-Rhman, 2020
*rmpA*	F-ACTGGGCTACCTCTGCTTCA R-CTTGCATGAGCCATCTTTCA	55	535	Liu and Guo, 2018
*rmpA2*	F-CTTTATGTGCAATAAG-GATGTT R-CCTCCTGGAGAGTAAGCATT	51	451	Liu and Guo, 2018
*magA*	F-GGTGCTCTTTACATCATTGC R-GCAATGGCCATTTGCGTTAG	54	1283	Liu and Guo, 2018
*iucA*	F-GCATAGGCGGATACGAACAT R-CACAGGGCAATTGCTTACCT	55	556	Liu and Guo, 2018
*peg-344*	F-CTTGAAACTATCCCTCCAGTC R-CCAGCGAAAGAATAACCCC	52	508	Teban-Man et al., 2021
*bla*_TEM_	F-CATTTCCGTGTCGCCCTTATTC R-CGTTCATCCATAGTTGCCTGAC	57	800	Fadare and Okoh, 2021
*bla*_SHV_	F-AGCCGCTTGAGCAAATTAAAC R-ATCCCGCAGATAAATCACCAC	58	713	Fadare and Okoh, 2021
*bla*_PER_	F-GCTCCGATAATGAAAGCGT R-TTCGGCTTGACTCGGCTGA	56	520	Fadare and Okoh, 2021
*bla*_VEB_	F-CATTTCCCGATGCAAAGCGT R-CGAAGTTTCTTTGGACTCTG	56	648	Fadare and Okoh, 2021
*bla*_FOX_	F-AACATGGGGTATCAGGGAGATG R-CAAAGCGCGTAACCGGATTGG	57	190	Khalifa et al., 2021
*bla*_DHA_	F-AACTTTCACAGGTGTGCTGGGT R-CCGTACGCATACTGGCTTTGC	58	405	Khalifa et al., 2021
*bla*_CMY_	F-GCTGCTCAAGGAGCACAGGAT R-CACATTGACATAGGTGTGGTGC	57	520	Khalifa et al., 2021
*bla*_LAT_	F-TGGCCAGAACTGACAGGCAAA R-TTTCTCCTGAACGTGGCTGGC	58	462	Khalifa et al., 2021
*bla*_ACT_	F-TCGGTAAAGCCGATGTTGCGG R-CTTCCACTGCGGCTGCCAGTT	59	302	Khalifa et al., 2021
*bla*_ACC_	F-AACAGCCTCAGCAGCCGGTTA R-TTCGCCGCAATCATCCCTAGC	53	346	Khalifa et al., 2021
*bla*_GES_	F-GTTTTGCAATGTGCTCAACG R-TGCCATAGCAATAGGCGTAG	53	371	Shao et al., 2021
*bla*_IMP_	F-TGAGCAAGTTATCTGTATTC R-TTAGTTGCTTGGTTTTGATG	47	740	Shao et al., 2021
*bla*_IMI_	F-CTACGCTTTAGACACTGGC R-AGGTTTCCTTTTCACGCTCA	52	482	Kolar et al., 2021

### Differentiation of hvKp

The hvKp isolates were differentiated from cKp based on phenotypic and genotypic criteria as previously described (Liu and Guo, 2018; Liao et al., 2020; Örsten et al., 2020; Serban et al., 2021; Zhu et al., 2021). Any isolate that showed positive results for one or more of the phenotypic or genotypic criteria was classified as hvKp. For phenotypic evaluation, the string test was performed. In this test, fresh colonies (24 h) of each isolate were tested on blood agar using a wire loop. Observation of a viscous string greater than 5 mm in length once the colony was taken with a loop wire was considered a positive test (Örsten et al., 2020). For genotypic analysis, *magA, rmpA, rmpA2, iucA*, and *peg344* were investigated by PCR using previously described primers (Table 1; Liu and Guo, 2018; Teban-Man et al., 2021). PCR and electrophoresis were performed similar to previous stage.

### Antibiotic susceptibility testing

A panel of different antibiotics, including amikacin (30 μg), ampicillin/sulbactam (10/10 μg), aztreonam (30 μg), cefazolin (30 μg), ceftriaxone (30 μg), ceftazidime (30 μg), cefotaxime (30 μg), cefoxitin (30 μg), cefepime (30 μg), chloramphenicol (30 μg), ciprofloxacin (5 μg), ertapenem (10 μg), gentamycin (10 μg), fosfomycin (200 μg), imipenem (10 μg), meropenem (10 μg), piperacillin/tazobactam (100/10 μg), tetracycline (30 μg), tobramycin (10 μg), and trimethoprim/sulfamethoxazole (1.25/23.75 μg) (Padtan Teb Co, Tehran, Iran and Mast Group Ltd, United Kingdom) were tested on Mueller–Hinton agar (MHA) (Merck, Darmstadt, Germany) using the Kirby-Bauer disc diffusion method according to Clinical Laboratory Standard Institute (CLSI) procedures (Clinical and Laboratory Standards Institute, 2021). Also, the minimum inhibitory concentrations (MICs) of imipenem, ertapenem, and meropenem were determined by the broth microdilution method following the criteria of CLSI 2021 (Clinical and Laboratory Standards Institute, 2021), and the MICs for tigecycline and colistin were determined according to the breakpoints of the European Committee on Antimicrobial Susceptibility Testing (EUCAST) 2021 (The European Committee on Antimicrobial Susceptibility Testing, 2021). Antibiotic powders were purchased from Sigma-Aldrich, Darmstadt, Germany. Isolates with an MIC ≥ 2 μg/mL for ertapenem and colistin, ≥4 μg/mL for meropenem and imipenem, and ≥0.5 μg/mL for tigecycline were considered resistant (Clinical and Laboratory Standards Institute, 2021; The European Committee on Antimicrobial Susceptibility Testing, 2021). The MIC_50_ and MIC_90_ of each antibiotic were defined as the lowest concentration of the antibiotic required to inhibit 50 and 90% of isolates, respectively (Vilaró et al., 2020). Any isolate that was resistant to one or more of the antibiotics imipenem, ertapenem, and meropenem was selected as carbapenem-resistant. Resistance to at least one antibiotic in three or more classes, resistance to at least one antibiotic in all but two or fewer classes, and resistance to all tested antibiotics in all classes were defined as MDR, extensively drug-resistant (XDR), and pandrug-resistant (PDR) isolates, respectively (Magiorakos et al., 2012). Multiple antibiotic resistance index (MARI) was measured as a/b, where a = the total number of antibiotics to which an isolate was resistant and b = the total number of antibiotics tested (Abdel-Rhman, 2020). *Escherichia coli* ATCC^®^ 25922™ was used as a quality control.

### Phenotypic detection and confirmation of extended-spectrum beta-lactamases

Based on the CLSI screening criteria, all *K. pneumoniae* isolates that had breakpoints of ≤27 mm for cefotaxime (30 μg), ≤22 mm for ceftazidime (30 μg), and ≤25 mm for ceftriaxone (30 μg), were selected for primary ESBL screening (Clinical and Laboratory Standards Institute, 2021). Subsequently, to confirm phenotypic ESBL detection, the CLSI-recommended combined disc test (CDT) was performed with cefotaxime (30 μg) and ceftazidime (30 μg) disks alone and in combination with clavulanic acid (30 μg/10 μg) (Clinical and Laboratory Standards Institute, 2021). ESBL producers were confirmed when an increase in zone diameter of cephalosporin/clavulanate of ≥5 mm was observed compared to cephalosporin alone. *E. coli* ATCC^®^ 25922™ and *K. pneumoniae* ATCC^®^ 700603™ were used as ESBL-negative and positive strains, respectively.

### Phenotypic detection and confirmation of the AmpC enzymes

The isolates that showed an inhibition zone of ≤18 mm against cefoxitin (30 μg) were considered as primary AmpC producers. These isolates were further confirmed by a combined disk method using a cefoxitin disk (30 μg) alone and in combination with phenylboronic acid (PBA) (400 μg) (Sigma-Aldrich, MO, USA). The cefoxitin disk and cefoxitin/PBA disk were dispensed onto an MHA plate that had already been inoculated with the test isolate (equivalent to 0.5 McFarland) by the standard disk diffusion method. Subsequently, the inoculated plates were incubated for 24 h at 37°C. The isolates were confirmed as AmpC producers when the zone diameter of cefoxitin/PBA increased by ≥5 mm compared to cefoxitin alone (Kuinkel et al., 2021).

### Phenotypic detection and confirmation of carbapenemases and metallo-beta-lactamases

According to CLSI guidelines, all carbapenem-resistant isolates were screened for carbapenemase and MBL production using the modified carbapenem inactivation method (mCIM) together with EDTA-modified carbapenem inactivation method (eCIM) (Clinical and Laboratory Standards Institute, 2021). For mCIM, carbapenem-resistant isolates (1 μl loopful) were dissolved in a microtube containing 2 ml of TSB medium. For eCIM, 1 μl loopful of carbapenem-resistant isolates were dissolved in a microtube containing 2 ml TSB and 20 μl of the 0.5 M EDTA (Sigma-Aldrich, MO, USA). A meropenem disk (10 μg) was placed in each tube, and both tubes were incubated at 35 ± 2°C for 4 h ± 15 min. The disks were then removed and placed on MHA plates freshly pre-inoculated with *E. coli* ATCC^®^ 25922™. After 18–24 h incubation at 35 ± 2°C, mCIM and eCIM results were interpreted as follows: carbapenemase positive (only mCIM positive): zone diameter of 6–15 mm, carbapenemase negative (mCIM negative): zone size ≥ 19 mm, MBL positive (both mCIM/eCIM positive): increase in eCIM zone size by ≥5 mm compared to zone size of mCIM (Clinical and Laboratory Standards Institute, 2021). It should be noted that eCIM was only valid for isolates that tested positive for mCIM. *E. coli* ATCC^®^ 25922™ and *K. pneumoniae* ATCC^®^ BAA-1705™ were used as negative and positive control strains, respectively.

### Molecular detection of extended-spectrum beta-lactamase, AmpC, carbapenemase, and metallo-beta-lactamases genes

Uniplex PCR was performed with specific primers to detect the presence of ESBLs (*bla*_CTX–M_, *bla*_SHV_, *bla*_TEM_, *bla*_PER_, and *bla*_VEB_), AmpC (*bla*_FOX_, *bla*_DHA_, *bla*_CMY_, *bla*_LAT_, *bla*_ACC_, and *bla*_ACT_), carbapenemase (*bla*_KPC_, *bla*_GES_, *bla*_OXA–48–like_, and *bla*_IMI_), and MBLs (*bla*_NDM_, *bla*_IMP_, and *bla*_VIM_) as previously described (Table 1; Maleki et al., 2018; Abdel-Rhman, 2020; Fadare and Okoh, 2021; Khalifa et al., 2021; Kolar et al., 2021; Shao et al., 2021). PCR and electrophoresis were performed in a similar manner to the previous steps. The annealing temperature and product size for each gene are listed in Table 1. The positive control genes were prepared from Pasteur Institute of Iran (Tehran, Iran).

### Enterobacterial repetitive intergenic consensus polymerase chain reaction

Enterobacterial repetitive intergenic consensus polymerase chain reaction (ERIC-PCR) was performed with the previously described primers in a final volume of 25 μl containing 1 μl of each primer, 3 μl of template DNA, 12.5 of master mix (Ampliqon, Denmark), and 7.5 μl of nuclease-free water using the Biorad S1000 thermocycler (USA) (Abdel-Rhman, 2020). Following set up was used for ERIC-PCR: an initial denaturation at 95°C for 5 min; 35 cycles of 1 min at 95°C, 1 min at 49°C, and 3 min at 72°C; and a final extension at 72°C for 7 min. Amplicons were separated by 2.0% agarose gel electrophoresis (80 V, 60 min) and recorded as TIFF images using a UV light transilluminator (Protein Simple, San Jose, CA, USA). Images were analyzed with BioNumerics software version 7.6.3 (Applied Maths, Kortrijk, Belgium). Unweighted-pair group method with arithmetic averages (UPGMA) and the Dice similarity coefficient were used to analyze the ERIC-PCR pattern dendrograms. A group of isolates that had a similarity coefficient of ≥90.0% was considered as one and the same cluster (Dalmolin et al., 2017).

### Statistical analysis

Descriptive analysis with mean, percentage, and frequency statistics was performed using Statistical Package for the Social Sciences (SPSS) version 22.0 (IBM Corporation, Armonk, NY, USA). Significant associations (*P*-value ≤ 0.05) of variables were evaluated with Chi-square and Fisher’s exact tests (Abdel-Rhman, 2020).

## Results

### Bacterial isolates

Using standard bacteriology tests, a total of 153 presumptive *K. pneumoniae* isolates were collected from different clinical samples of 82 (53.6%) males and 71 females (46.4%). All isolates showed the 130 bp band of the *16S–23S ITS* gene in PCR and were confirmed as *K. pneumonia*e. The mean ± SD age of patients was 43.1 ± 16.3 (10–81 years) for males and 36.9 ± 12.7 (11–82 years) for females. Using phenotypic and genotypic criteria, 136 (88.9%) and 17 (11.1%) isolates were identified as cKp and hvKp, respectively. Of all hvKp isolates, 6 (35.3%), 17 (100.0%), and 6 (35.3%) were positive for the string test, gene markers, and both, respectively. The occurrence of hvKp gene markers was as follows: *magA* (11.8%, *n* = 2), *rmpA* (11.8%, *n* = 2), *rmpA2* (52.9%, *n* = 9), *iucA* (52.9%, *n* = 9), and *peg344* (35.3%, *n* = 6). The co-occurrence of two or more markers was detected in 5 (29.4%) isolates as follows: *magA*/*rmpA2* (*n* = 1), *rmpA2*/*iucA*/*peg344* (*n* = 3), and *magA*/*rmpA*/*rmpA2*/*iucA*/*peg344* (*n* = 1).

### Antibiotic resistance rates of cKp, hvKp, CR-cKp, and CR-hvKp

Using the carbapenem antibiotic disk diffusion test and the broth microdilution method, the overall resistance to carbapenems was 54.2% (*n* = 83/153), including 90.4% (*n* = 75/83) cKp and 9.6% (*n* = 8/83) hvKp isolates. In other words, 55.1% (*n* = 75/136) of cKp and 47.1% (*n* = 8/17) of hvKp isolates were CR-cKp and CR-hvKp, respectively. Imipenem was the most effective carbapenem against *K. pneumoniae* isolates, with a susceptibility rate of 48.4% (*n* = 74/153), followed by ertapenem (47.7%, *n* = 73/153), and meropenem (45.8%, *n* = 70/153). Of 75 CR-cKp isolates, 60 (80.0%), 8 (10.7%), and 7 (9.3%) isolates were resistant to ertapenem/imipenem/meropenem, ertapenem/meropenem, and imipenem/meropenem, respectively. However, all CR-hvKp isolates were simultaneously resistant to meropenem/imipenem/ertapenem. The carbapenem-resistant isolates had MICs ranging from 0.03 to 64 μg/mL, MIC_50_ = 8 μg/mL, MIC_90_ = 32 μg/mL for ertapenem; MICs ranging from 0.03 to 64 μg/mL, MIC_50_ = 16 μg/mL, MIC_90_ = 32 μg/mL for imipenem; and MICs ranging from 8 to 64 μg/mL, MIC_50_ = 16 μg/mL, MIC_90_ = 32 μg/mL for meropenem. The detailed antibiotic resistance rates of cKp, hvKp, CR-cKp, and CR-hvKp isolates were summarized in Table 2. All isolates showed the highest susceptibility to colistin (98.0%, *n* = 150/153), tetracycline (89.5%, *n* = 137/153), and tigecycline (80.4%, *n* = 123/153) and the highest resistance to ampicillin/sulbactam (100.0%, *n* = 153/153). Resistance rates to other antibiotics ranged from 29.4% (*n* = 45/153) for aztreonam to 64.7% (*n* = 99/153) for cefazolin. CR-cKp and CR-hvKp isolates showed resistance rates of more than 50.0% against gentamicin, tobramycin, amikacin, and piperacillin/tazobactam. Also, all hvKp isolates (*n* = 17) including CR-hvKp (*n* = 8) strains were susceptible to colistin, tigecycline, and tetracycline. There were no significant differences (*P*-value ≤ 0.05) in the antibiotic resistance patterns of the cKp with hvKp isolates and the CR-cKp with CR-hvKp isolates except for tigecycline (Table 2). The carbapenem-resistant isolates had MICs ranging from 8 to 64 μg/mL, MIC_50_ = 16 μg/mL, MIC_90_ = 32 μg/mL for tigecycline; and MICs ranging from 0.03 to 4 μg/mL, MIC_50_ = 0.06 μg/mL, MIC_90_ = 0.25 μg/mL for colistin. In total, 64.1% (*n* = 98/153) of all isolates were resistant against third-generation cephalosporins. Of 136 cKp, 1 (0.7%), 1 (0.7%), 1 (0.7%), 3 (2.2%), and 81 (59.6%) isolates were resistant to cefotaxime, cefotaxime/ceftazidime, ceftazidime/ceftriaxone, cefotaxime/ceftriaxone, and cefotaxime/ceftazidime/ceftriaxone, respectively. Of 17 hvKp, 11 (64.7%) isolates were simultaneously resistant to cefotaxime/ceftazidime/ceftriaxone. Of 75 CR-cKp isolates, 69 (92.0%) strains showed resistance against third-generation cephalosporin as follows: cefotaxime (1, 1.3%), ceftazidime/ceftriaxone (1, 1.3%), cefotaxime/ceftazidime (1, 1.3%), cefotaxime/ceftriaxone (3, 4.0%), and cefotaxime/ceftazidime/ceftriaxone (63, 84.0%). Also, all CR-hvKp were simultaneously resistant to third-generation cephalosporins. In total, 66.7% (*n* = 102/153) and 7.8% (*n* = 12/153) of isolates were MDR and XDR, respectively. None of the isolates were PDR. Of 75 CR-cKp, 84.0% (*n* = 63) and 16.0% (*n* = 12) were MDR and XDR, respectively. While, all CR-hvKp (100.0%, *n* = 8) isolates were MDR (Table 2). The 83 carbapenem-resistant *K. pneumoniae* isolates had 57 (A1–A57) different antibiotypes (antibiotic resistance patterns) (Table 3). A2 (12.0%, *n* = 10), A4 (7.2%, *n* = 6), and A5 (7.2%, *n* = 6) were the most frequent patterns. Also, MARI ranged from 0.1 to 1.0 and the majority of isolates (84.3%, *n* = 70) had MARI of ≥0.5.

**TABLE 2:** Antibiotic resistance rates of classic *Klebsiella pneumoniae* (cKp), hypervirulent *K. pneumoniae* (hvKp), carbapenem-resistant classic *K. pneumoniae* (CR-cKp), and carbapenem-resistant hypervirulent *K. pneumoniae* (CR-hvKp) isolates.

Antibiotics	Total *Klebsiella* isolates *n*: 153			cKp *n*: 136			hvKp *n*: 17			*P*-value	Total carbapenem-resistant *Klebsiella* isolates *n*: 83			CR-cKp *n* : 75			CR*-*hvKp *n*: 8			*P*-value
	R	I	S	R	I	S	R	I	S		R	I	S	R	I	S	R	I	S	
	*n* (%)			*n* (%)			*n* (%)				*n* (%)			*n* (%)			*n* (%)			
Gentamicin	80 (52.3)	0 (0.0)	73 (47.7)	72 (52.9)	0 (0.0)	64 (47.1)	8 (47.1)	0 (0.0)	9 (52.9)	0.798	55 (66.3)	0 (0.0)	28 (33.7)	50 (66.6)	0 (0.0)	25 (33.3)	5 (62.5)	0 (0.0)	3 (37.5)	>0.999
Tobramycin	85 (55.6)	1 (0.7)	67 (43.8)	75 (55.1)	1 (0.7)	60 (44.1)	10 (58.8)	0 (0.0)	7 (41.2)	>0.803	60 (72.3)	1 (1.2)	22 (26.5)	53 (70.7)	1 (1.3)	21 (28.0)	7 (87.5)	0 (0.0)	1 (12.5)	0.676
Amikacin	63 (41.2)	5 (3.2)	85 (55.6)	55 (40.4)	4 (2.9)	77 (56.6)	8 (47.1)	1 (5.8)	8 (47.1)	>0.611	57 (68.7)	4 (4.8)	22 (26.5)	50 (66.7)	4 (5.3)	21 (28.0)	7 (87.5)	0 (0.0)	1 (12.5)	0.432
Piperacillin-tazobactam	53 (34.6)	0 (0.0)	100 (65.4)	46 (33.8)	0 (0.0)	90 (66.2)	7 (41.2)	0 (0.0)	10 (58.8)	0.594	52 (62.7)	0 (0.0)	31 (37.3)	45 (60.0)	0 (0.0)	30 (40.0)	7 (87.5)	0 (0.0)	1 (12.5)	0.248
Ertapenem	76 (49.7)	5 (3.3)	73 (47.7)	68 (50.0)	4 (2.9)	64 (47.1)	8 (47.1)	1 (5.8)	8 (47.1)	>0.999	76 (91.6)	4 (4.8)	3 (3.6)	68 (90.7)	4 (5.3)	3 (4.0)	8 (100.0)	0 (0.0)	0 (0.0)	>0.999
Imipenem	75 (49.0)	4 (2.6)	74 (48.4)	67 (49.3)	4 (2.9)	65 (47.8)	8 (47.1)	0 (0.0)	9 (52.9)	>0.999	75 (90.4)	4 (4.8)	4 (4.8)	67 (89.3)	4 (5.3)	4 (5.3)	8 (100.0)	0 (0.0)	0 (0.0)	>0.999
Meropenem	83 (54.2)	0 (0.0)	70 (45.8)	74 (54.4)	0 (0.0)	62 (45.6)	9 (52.9)	0 (0.0)	8 (47.1)	>0.999	83 (100.0)	0 (0.0)	0 (0.0)	75 (100.0)	0 (0.0)	0 (0.0)	8 (100.0)	0 (0.0)	0 (0.0)	>0.999
Cefazolin	99 (64.7)	3 (0.2)	51 (33.3)	88 (64.7)	2 (1.5)	46 (33.8)	11 (64.7)	1 (5.8)	5 (29.4)	>0.999	78 (94.0)	3 (3.6)	2 (2.4)	70 (93.3)	3 (4.0)	2 (2.7)	8 (100.0)	0 (0.0)	0 (0.0)	>0.999
Cefoxitin	73 (47.7)	6 (3.9)	74 (48.4)	65 (47.8)	5 (3.7)	66 (48.5)	8 (47.1)	1 (5.8)	8 (47.1)	>0.999	63 (75.9)	4 (4.8)	16 (19.3)	56 (74.7)	3 (4.0)	16 (21.3)	7 (87.5)	1 (12.5)	0 (0.0)	0.334
Cefotaxime	97 (63.4)	2 (1.3)	54 (35.3)	86 (63.2)	2 (3.7)	48 (35.3)	11 (64.7)	0 (0.0)	6 (35.3)	>0.999	76 (91.6)	2 (2.4)	5 (6.0)	68 (90.7)	2 (2.7)	5 (6.7)	8 (100.0)	0 (0.0)	0 (0.0)	>0.999
Ceftazidime	94 (61.4)	3 (2.0)	56 (36.6)	83 (61.0)	3 (2.2)	50 (36.8)	11 (64.7)	0 (0.0)	6 (35.3)	>0.999	73 (88.0)	3 (3.6)	7 (8.4)	65 (86.7)	3 (4.0)	7 (9.3)	8 (100.0)	0 (0.0)	0 (0.0)	>0.999
Ceftriaxone	95 (62.1)	1 (0.7)	57 (37.3)	84 (61.8)	1 (0.7)	51 (37.5)	11 (64.7)	0 (0.0)	6 (35.3)	>0.999	75 (90.4)	0 (0.0)	8 (9.6)	67 (89.3)	0 (0.0)	8 (10.7)	8 (100.0)	0 (0.0)	0 (0.0)	>0.999
Cefepime	93 (60.8)	1 (0.7)	59 (38.6)	82 (60.3)	1 (0.7)	53 (39.0)	11 (64.7)	0 (0.0)	6 (35.3)	0.799	73 (88.0)	1 (1.2)	9 (10.8)	65 (86.7)	1 (1.3)	9 (12.0)	8 (100.0)	0 (0.0)	0 (0.0)	0.589
Ciprofloxacin	95 (62.1)	4 (2.6)	54 (35.3)	83 (61.0)	4 (2.9)	49 (36.0)	12 (70.6)	0 (0.0)	5 (29.4)	0.603	68 (81.9)	4 (4.8)	11 (13.3)	60 (80.0)	4 (5.3)	11 (14.7)	8 (100.0)	0 (0.0)	0 (0.0)	0.591
Trimethoprim/ sulfamethoxazole	92 (60.1)	0 (0.0)	61 (39.9)	81 (59.6)	0 (0.0)	55 (40.4)	11 (70.6)	0 (0.0)	5 (29.4)	0.593	63 (75.9)	0 (0.0)	20 (24.1)	56 (74.7)	0 (0.0)	19 (25.3)	7 (87.5)	0 (0.0)	1 (12.5)	0.673
Tigecycline	30 (19.6)	0 (0.0)	123 (80.4)	30 (22.1)	0 (0.0)	106 (77.9)	0 (0.0)	0 (0.0)	17 (100.0)	0.0254*	27 (32.5)	0 (0.0)	56 (67.5)	27 (36.0)	0 (0.0)	48 (64.0)	0 (0.0)	0 (0.0)	8 (100.0)	0.049*
Aztreonam	45 (29.4)	0 (0.0)	108 (70.6)	38 (27.9)	0 (0.0)	98 (72.1)	7 (41.2)	0 (0.0)	10 (58.8)	0.269	44 (53.0)	0 (0.0)	39 (47.0)	37 (49.3)	0 (0.0)	38 (50.7)	7 (87.5)	0 (0.0)	1 (12.5)	0.061
Ampicillin/ sulbactam	153 (100.0)	0 (0.0)	0 (0.0)	136 (100.0)	0 (0.0)	0 (0.0)	17 (100.0)	0 (0.0)	0 (0.0)	>0.999	83 (100.0)	0 (0.0)	0 (0.0)	75 (100.0)	0 (0.0)	0 (0.0)	8 (100.0)	0 (0.0)	0 (0.0)	>0.999
Chloramphenicol	63 (41.2)	3 (2.0)	87 (56.9)	58 (42.6)	2 (1.5)	76 (55.9)	5 (29.4)	1 (5.8)	11 (64.7)	0.429	54 (65.1)	3 (3.6)	26 (31.3)	51 (68.0)	3 (4.0)	21 (28.0)	3 (37.5)	0 (0.0)	5 (62.5)	0.105
Colistin	3 (2.0)	0 (0.0)	150 (98.0)	3 (2.2)	0 (0.0)	133 (97.8)	0 (0.0)	0 (0.0)	17 (100.0)	>0.999	3 (3.6)	0 (0.0)	80 (96.4)	3 (4.0)	0 (0.0)	72 (96.0)	0 (0.0)	0 (0.0)	8 (100.0)	>0.999
Tetracycline	16 (10.5)	0 (0.0)	137 (89.5)	16 (11.8)	0 (0.0)	120 (88.2)	0 (0.0)	0 (0.0)	17 (100.0)	0.219	16 (19.3)	0 (0.0)	67 (80.7)	16 (21.3)	0 (0.0)	59 (78.7)	0 (0.0)	0 (0.0)	8 (100.0)	0.344
Fosfomycin	53 (34.6)	0 (0.0)	100 (65.4)	50 (36.8)	0 (0.0)	86 (63.2)	3 (17.6)	0 (0.0)	14 (82.4)	0.176	44 (53.0)	0 (0.0)	39 (47.0)	37 (49.3)	0 (0.0)	38 (50.7)	7 (87.5)	0 (0.0)	1 (12.5)	0.061
MDR	102 (66.7)			90 (66.2)			12 (70.6)			0.792	71 (85.5)			63 (84.0)			8 (100.0)			0.595
XDR	12 (7.8)			12 (8.8)			0 (0.0)			0.363	12 (14.5)			12 (16.0)			0 (0.0)			0.595

R, resistant; I, intermediate; S, susceptible; MDR, multidrug-resistant; XDR, extensively drug-resistant.

*Significant association.

**TABLE 3:** Antibiotypes (antibiotic resistance patterns) of 83 carbapenem-resistant *Klebsiella pneumoniae* isolates.

Antibiotypes	Number of antibiotics	MARI	Isolates *n* (%)	Resistance patterns
A1	23	1	1 (1.2)	GEN-TN-AN-PTZ-ERT-IMI-MER-CZ-FOX-CTX-CAZ-CRO-CPM-CIP-SXT-TGC-AZM-AMP-SAM-CL-CO-FOS-TET
A2	22	0.9	10 (12.0)	GEN-TN-AN-PTZ-ERT-IMI-MER-CZ-FOX-CTX-CAZ-CRO-CPM-CIP-SXT-TGC-AZM-AMP-SAM-CL-FOS-TET
A3			1 (1.2)	GEN-TN-AN–PTZ-ERT-IMI-MER-CZ-CTX-CAZ-CRO-CPM-CIP-SXT-TGC-AZM-AMP-SAM-CL-CO-FOS-TET
A4	21	0.9	6 (7.2)	GEN-TN-AN-PTZ-ERT-IMI-MER-CZ-FOX-CTX-CAZ-CRO-CPM-CIP-SXT-TGC-AZM-AMP-SAM-CL-FOS
A5	20	0.8	6 (7.2)	GEN-TN-AN-PTZ-ERT-IMI-MER-CZ-FOX-CTX-CAZ-CRO-CPM-CIP-SXT-AZM-AMP-SAM-CL-FOS
A6			1 (1.2)	GEN-TN-AN-PTZ-ERT-IMI-MER-CZ-FOX-CTX-CAZ-CRO-CPM-CIP-TGC-AZM-AMP-SAM-CL-FOS
A7			1 (1.2)	GEN-TN-AN-PTZ-ERT-IMI-MER-CZ-FOX-CTX-CAZ-CRO-CPM-CIP-SXT-TGC-AMP-SAM-CL-TET
A8			3 (3.6)	GEN-TN-AN-PTZ-ERT-IMI-MER-CZ-FOX-CTX-CAZ-CRO-CPM-CIP-SXT-TGC-AZM-AMP-SAM-FOS
A9	19		1 (1.2)	GEN-TN-AN-PTZ-ERT-MER-CZ-FOX-CTX-CAZ-CRO-CPM-CIP-SXT-AZM-AMP-SAM-CL-FOS
A10			1 (1.2)	GEN-TN-AN-PTZ-ERT-IMI-MER-CZ-FOX-CTX-CAZ-CRO-CPM-CIP-AZM-AMP-SAM-CL-FOS
A11			1 (1.2)	GEN-TN-AN-PTZ-ERT-IMI-MER-CZ-FOX-CTX-CAZ-CRO-CPM-CIP-SXT-AZM-AMP-SAM-CL
A12			4 (4.8)	GEN-TN-AN-PTZ-ERT-IMI-MER-CZ-FOX-CTX-CAZ-CRO-CPM-CIP-SXT-AZM-AMP-SAM-FOS
A13			1 (1.2)	GEN-TN-AN-PTZ-ERT-IMI-MER-CZ-FOX-CTX-CAZ-CRO-CPM-CIP-SXT-TGC-AMP-SAM-CL
A14	18	0.7	1 (1.2)	GEN-TN-AN-ERT-IMI-MER-CZ-FOX-CTX-CAZ-CRO-CPM-CIP-SXT-AMP-SAM-CL-CO
A15			1 (1.2)	GEN-TN-AN-PTZ-ERT-IMI-MER-CZ-FOX-CTX-CAZ-CRO-CPM-CIP-AZM-AMP-SAM-FOS
A16			1 (1.2)	GEN-TN-AN-PTZ-ERT-MER-CZ-FOX-CTX-CAZ-CRO-CPM-CIP-SXT-AMP-SAM-CL-TET
A17			1 (1.2)	GEN-AN-PTZ-ERT-IMI-MER-CZ-FOX-CTX-CAZ-CRO-CPM-CIP-SXT-AZM-AMP-SAM-FOS
A18			1 (1.2)	TN-AN-PTZ-ERT-IMI-MER-CZ-FOX-CTX-CAZ-CRO-CPM-CIP-SXT-AZM-AMP-SAM-FOS
A19			2 (2.4)	GEN-TN-AN-PTZ-ERT-IMI-MER-CZ-FOX-CTX-CAZ-CRO-CPM-CIP-SXT-AMP-SAM-CL
A20	17		1 (1.2)	GEN-TN-AN-PTZ-ERT-IMI-MER-CZ-FOX-CTX-CAZ-CRO-CPM-CIP-AMP-SAM-CL
A21			1 (1.2)	GEN-TN-PTZ-ERT-IMI-MER-CZ-CTX-CAZ-CRO-CPM-CIP-SXT-AZM-AMP-SAM-FOS
A22			1 (1.2)	TN-AN-ERT-IMI-MER-CZ-FOX-CTX-CAZ-CRO-CPM-CIP-SXT-AZM-AMP-SAM-FOS
A23			1 (1.2)	GEN-TN-ERT-IMI-MER-CZ-CTX-CAZ-CRO-CPM-CIP-SXT-AZM-AMP-SAM-CL-FOS
A24	16	0.6	1 (1.2)	GEN-TN-ERT-IMI-MER-CZ-FOX-CTX-CAZ-CRO-CPM-CIP-SXT-AMP-SAM-CL
A25			2 (2.4)	GEN-TN-AN-IMI-MER-CZ-FOX-CTX-CAZ-CRO-CPM-CIP-SXT-AMP-SAM-CL
A26			1 (1.2)	GEN-AN-ERT-IMI-MER-CZ-FOX-CTX-CAZ-CRO-CPM-CIP-SXT-AMP-SAM-CL
A27			1 (1.2)	GEN-TN-AN-IMI-MER-CZ-FOX-CTX-CAZ-CRO-CPM-CIP-SXT-AMP-SAM-FOS
A28	15		1 (1.2)	GEN-TN-ERT-IMI-MER-CZ-CTX-CAZ-CRO-CPM-CIP SXT-AMP-SAM-CL
A29			1 (1.2)	TN-ERT-IMI-MER-CZ-FOX-CTX-CAZ-CRO-CPM-CIP-SXT-AZM-AMP-SAM
A30			1 (1.2)	TN-ERT-IMI-MER-CZ-FOX-CTX-CAZ-CRO-CPM-CIP-SXT-AMP-SAM-CL
A31			1 (1.2)	TN-PTZ-ERT-MER-CZ-FOX-CTX-CAZ-CRO-CPM-CIP-SXT-TGC-AMP-SAM
A32			1 (1.2)	GEN-TN-AN-ERT-IMI-MER-CZ-FOX-CAZ-CRO-CPM-CIP-SXT-AMP-SAM
A33			1 (1.2)	AN-PTZ-ERT-IMI-MER-CZ-FOX-CTX-CAZ-CRO-CPM-AZM-AMP-SAM-FOS
A34	14		1 (1.2)	AN-PTZ-ERT-IMI-MER-CZ-FOX-CTX-CAZ-CRO-CPM-CIP-AMP-SAM
A35			1 (1.2)	TN-ERT-IMI-MER-CZ-FOX-CTX-CAZ-CRO-CPM-SXT-AMP-SAM-CL
A36			1 (1.2)	GEN-TN-ERT-MER-CZ-CTX-CAZ-CRO-CPM-CIP-SXT-AMP-SAM-FOS
A37			1 (1.2)	GEN-TN-AN-ERT-IMI-MER-CZ-CTX-CAZ-CRO-CPM-AMP-SAM-CL
A38	13	0.5	1 (1.2)	PTZ-ERT-IMI-MER-CZ-FOX-CTX-CAZ-CRO-CIP-SXT-AMP-SAM
A39	12		1 (1.2)	ERT-IMI-MER-CZ-CTX-CAZ-CRO-CPM-SXT-AMP-SAM-TET
A40			1 (1.2)	AN-ERT-MER-CZ-CTX-CPM-SXT-AZM-AMP-SAM-CL-FOS
A41			1 (1.2)	PTZ-IMI-MER-CZ-FOX-CTX-CAZ-CRO-CPM-AMP-SAM-CL
A42			1 (1.2)	IMI-MER-CZ-CTX-CAZ-CRO-CPM-CIP-SXT-AMP-SAM-CL
A43			1 (1.2)	AN-ERT-MER-CZ-FOX-CTX-CAZ-CRO-CPM-AMP-SAM-CL
A44			1 (1.2)	TN-ERT-IMI-MER-CZ-CTX-CAZ-CRO-CPM-SXT-AMP-SAM
A45	11	0.4	1 (1.2)	PTZ-ERT-IMI-MER-CZ-FOX-CTX-CAZ-CIP-AMP-SAM
A46			1 (1.2)	PTZ-ERT-IMI-MER-CZ-FOX-CTX-CRO-CIP-AMP-SAM
A47			1 (1.2)	ERT-IMI-MER-CZ-CTX-CAZ-CRO-CIP-SXT-AMP-SAM
A48			1 (1.2)	ERT-MER-CZ-CTX-CAZ-CRO-CPM-SXT-AMP-SAM-TET
A49	10		1 (1.2)	ERT-IMI-MER-CTX-CRO-CPM-CIP-AMP-SAM-CL
A50			1 (1.2)	AN-ERT-MER-CZ-CTX-CRO-CPM-AMP-SAM-CL
A51			1 (1.2)	ERT-IMI-MER-CZ-FOX-CPM-CIP-AMP-SAM-CL
A52	9	0.3	1 (1.2)	ERT-IMI-MER-CZ-CTX-CAZ-CRO-AMP-SAM
A53	7		1 (1.2)	ERT-IMI-MER-CIP-AMP-SAM-CL
A54			1 (1.2)	ERT-IMI-MER-CZ-AMP-SAM-CL
A55			1 (1.2)	ERT-IMI-MER-AMP-TGC-SAM-CL
A56	5	0.2	1 (1.2)	IMI-MER-TGC-AMP-SAM
A57	4	0.1	1 (1.2)	IMI-MER-AMP-SAM

AN, amikacin; SAM, ampicillin/sulbactam; AZM, aztreonam; CZ, cefazolin; CRO, ceftriaxone; CAZ, ceftazidime; CTX, cefotaxime; FOX, cefoxitin; CPM, cefepime; CL, chloramphenicol; CIP, ciprofloxacin; ERT, ertapenem; GEN, gentamycin; FOS, fosfomycin; MER, meropenem; PTZ, piperacillin/tazobactam; TET, tetracycline; TN, tobramycin; SXT, trimethoprim/sulfamethoxazole; TGC, tigecycline; CO, colistin; MARI, multiple antibiotic resistance indexes.

### Distribution of cKp, hvKp, CR-cKp, and CR-hvKp

The distribution of cKp, hvKp, CR-cKp, and CR-hvKp isolates according to hospital, age, gender, samples, and wards was presented in Table 4. The highest distribution of cKp isolates was observed in Golestan Hospital (52.2%, *n* = 71/136), male patients (50.7%, *n* = 69/136), patients aged 26–41 years (39.0%, *n* = 53/136), urine samples (50.0%, *n* = 68/136), and men ward (27.9%, *n* = 38/136). Meanwhile, the highest occurrence of hvKp isolates was found in Golestan Hospital (64.7%, *n* = 11/17), male patients (76.5%, *n* = 13/17), patients aged 42–57 years (35.3%, *n* = 6/17), urine samples (64.7%, *n* = 11/17), and men ward (52.9%, *n* = 9/17) (Table 4). Although the distribution of cKp and hvKp was not significantly different according to the hospital, age, gender, sample type, and various wards, the frequency of hvKp in the men ward was significantly higher than that of cKp (*P*-value = 0.049). Similar results were obtained for CR-cKp and CR-hvKp isolates (Table 4). Nevertheless, the CR-cKp isolates were more frequent in females (54.7%, *n* = 41/75) than in males (45.3%, *n* = 34/75). Also, the CR-hvKp isolates were more prevalent in intensive care unit (ICU) (37.5%, *n* = 3/8) than other wards. However, the distribution of CR-cKp and CR-hvKp was not significantly different according to the various items (Table 4).

**TABLE 4:** The frequency of classic *Klebsiella pneumoniae* (cKp), hypervirulent *K. pneumoniae* (hvKp), carbapenem-resistant classic *K. pneumoniae* (CR-cKp), and carbapenem-resistant hypervirulent *K. pneumoniae* (CR-hvKp) isolates according to hospitals, ages, genders, samples, and wards.

Isolates (*n* = 153) *n* (%)	cKp (*n* = 136) *n* (%)	hvKp (*n* = 17) *n* (%)	*P*-value	Carbapenem-resistant isolates (*n* = 83) *n* (%)	CR-cKp (*n* = 75) *n* (%)	CR-hvKp (*n* = 8) *n* (%)	*P*-value
**Hospitals**				**Hospitals**			
Imam Khomeini 48 (31.4)	43 (31.6)	5 (29.4)	>0.999	Imam Khomeini 25 (30.1)	22 (29.3)	3 (37.5)	0.692
Golestan 82 (53.6)	71 (52.2)	11 (64.7)	0.441	Golestan 44 (53.0)	39 (52.0)	5 (62.5)	0.717
Razi 15 (9.8)	14 (10.3)	1 (5.9)	>0.999	Razi 6 (7.2)	6 (8.0)	0 (0.0)	>0.999
Taleghani 8 (5.2)	8 (5.9)	0 (0.0)	0.599	Taleghani 8 (9.6)	8 (10.7)	0 (0.0)	>0.999
**Genders**				**Genders**			
Males 82 (53.6)	69 (50.7)	13 (76.5)	0.069	Males 40 (48.2)	34 (45.3)	6 (75.0)	0.147
Females 71 (46.4)	67 (49.3)	4 (23.5)	0.069	Females 43 (51.8)	41 (54.7)	2 (25.5)	0.147
**Ages (year)**				**Ages (year)**			
10–25 25 (16.3)	22 (16.2)	3 (17.6)	>0.999	10–25 15 (18.1)	15 (20.0)	0 (0.0)	0.34
26–41 58 (37.9)	53 (39.0)	5 (29.4)	0.598	26–41 35 (42.2)	33 (44.0)	2 (25.0)	0.458
42–57 52 (37.9)	46 (33.8)	6 (35.3)	>0.999	42–57 24 (28.9)	20 (26.7)	4 (50.0)	0.220
58–73 13 (37.9)	10 (7.4)	3 (17.6)	0.161	58–73 6 (7.2)	4 (5.3)	2 (25.0)	0.101
>73 5 (3.3)	5 (3.7)	0 (0.0)	>0.999	>73 3 (3.6)	3 (4.0)	0 (0.0)	>0.999
**Samples**				**Samples**			
Urine 79 (51.6)	68 (50.0)	11 (64.7)	0.309	Urine 35 (42.2)	32 (42.7)	3 (37.5)	>0.999
Blood 35 (22.9)	33 (24.3)	2 (11.8)	0.363	Blood 16 (19.3)	15 (20.0)	1 (12.5)	>0.999
Tracheal tube 5 (3.3)	3 (2.2)	2 (11.8)	0.095	Tracheal tube 5 (6.0)	3 (4.0)	2 (25.0)	0.071
Wound 12 (7.8)	11 (8.1)	1 (5.9)	>0.999	Wound 11 (13.3)	10 (13.3)	1 (12.5)	>0.999
Abscess 1 (3.3)	1 (0.7)	0 (0.0)	>0.999	Abscess 1 (1.2)	1 (1.3)	0 (0.0)	>0.999
Peritoneal fluid 7 (4.6)	7 (5.1)	0 (0.0)	>0.999	Peritoneal fluid 5 (6.0)	5 (6.7)	0 (0.0)	>0.999
Sputum 6 (3.9)	5 (3.7)	1 (5.9)	0.513	Sputum 6 (7.2)	5 (6.7)	1 (12.5)	0.467
Synovial fluid 6 (3.9)	6 (4.4)	0 (0.0)	>0.999	Synovial fluid 2 (2.4)	2 (2.7)	0 (0.0)	>0.999
Tonsil 2 (1.3)	2 (1.5)	0 (0.0)	>0.999	Tonsil 2 (2.4)	2 (2.7)	0 (0.0)	>0.999
**Wards**				**Wards**			
Internal medicine 16 (10.5)	14 (10.3)	2 (11.8)	0.693	Internal medicine 7 (8.4)	6 (8.0)	1 (12.5)	0.522
Intensive care unit 11 (7.2)	8 (5.9)	3 (17.6)	0.107	Intensive care unit 11 (13.3)	8 (10.7)	3 (37.5)	0.068
Women 29 (19.0)	27 (19.9)	2 (11.8)	0.531	Women 9 (10.8)	8 (10.7)	1 (12.5)	>0.999
Men 47 (30.7)	38 (27.9)	9 (52.9)	0.049*	Men 19 (22.9)	17 (22.7)	2 (25.0)	>0.999
Surgery 9 (30.7)	8 (5.9)	1 (5.9)	>0.999	Surgery 5 (6.0)	4 (5.3)	1 (12.5)	0.406
Burn 8 (5.2)	8 (5.9)	0 (0.0)	0.599	Burn 8 (9.6)	8 (10.7)	0 (0.0)	>0.999
Infectious diseases 5 (3.3)	5 (3.7)	0 (0.0)	>0.999	Infectious diseases 4 (4.8)	4 (5.3)	0 (0.0)	>0.999
Urology 9 (30.7)	9 (6.6)	0 (0.0)	0.598	Urology 9 (10.8)	9 (12.0)	0 (0.0)	0.589
Emergency 7 (4.6)	7 (5.1)	0 (0.0)	>0.999	Emergency 2 (2.4)	2 (2.7)	0 (0.0)	>0.999
Nephrology 1 (0.7)	1 (0.7)	0 (0.0)	>0.999	Nephrology 1 (1.2)	1 (1.3)	0 (0.0)	>0.999
Neurology 2 (1.3)	2 (1.5)	0 (0.0)	>0.999	Neurology 2 (2.4)	2 (2.7)	0 (0.0)	>0.999
Orthopedy 5 (3.3)	5 (3.7)	0 (0.0)	>0.999	Orthopedy 2 (2.4)	2 (2.7)	0 (0.0)	>0.999
Pediatrics 2 (1.3)	2 (1.5)	0 (0.0)	>0.999	Pediatrics 2 (2.4)	2 (2.7)	0 (0.0)	>0.999
Ear, Nose, Throat 2 (1.3)	2 (1.5)	0 (0.0)	>0.999	Ear, Nose, Throat 2 (2.4)	2 (2.7)	0 (0.0)	>0.999

*Significant association.

### Phenotypic and genotypic prevalence of extended-spectrum beta-lactamases in CR-cKp and CR-hvKp

Using third-generation cephalosporin resistance criteria, 92.0% (*n* = 69/75) and 100.0% (*n* = 8/8) of CR-cKp and CR-hvKp were presumptive ESBL producers, respectively (Table 5). However, the confirmatory CDT method showed that 26.7% (*n* = 20/75) and 12.5% (*n* = 1/8) of CR-cKp and CR-hvKp were ESBL producers, respectively. All CDT positive isolates were also positive for at least one ESBL gene by PCR. The distribution of ESBL genes among CR-cKp and CR-hvKp was as follows: for CR-cKp: *bla*_CTX–M_ (26.7%, *n* = 20/75), *bla*_SHV_ (24.0%, *n* = 18/75), *bla*_TEM_ (10.7%, *n* = 8/75); and for CR-hvKp: *bla*_CTX–M_ (12.5%, *n* = 1/8), *bla*_SHV_ (12.5%, *n* = 1/8), and *bla*_TEM_ (0.0%, *n* = 0/8) (Table 5). None of the isolates were positive for *bla*_PER_ and *bla*_VEB_ genes. The result of CDT phenotypic confirmatory test was consistent with the PCR assay. Moreover, the distribution of ESBLs was not significantly different in CR-cKp and CR-hvKp isolates (*P*-value > 0.05, Table 5).

**TABLE 5:** Prevalence of beta-lactamase determinants and their genotypes in carbapenem-resistant classic *Klebsiella pneumoniae* (CR-cKp) and carbapenem-resistant hypervirulent *K. pneumoniae* (CR-hvKp) isolates.

Beta-lactamase determinants	Total carbapenem-resistant *Klebsiella pneumoniae* isolates *n*: 83	CR-cKp *n*: 75	CR-hvKp *n*: 8	*P*-value
	*n* (%)	*n* (%)	*n* (%)	
**ESBLs**				
Screening of ESBLs using third-generation cephalosporin resistance criteria	77 (92.8)	69 (92.0)	8 (100.0)	>0.999
Phenotypic ESBLs confirmation using CDT test	21 (25.3)	20 (26.7)	1 (12.5)	0.673
Genotypic ESBLs confirmation using PCR	21 (25.3)	20 (26.7)	1 (12.5)	0.673
*bla*_CTX–M_	21 (25.3)	20 (26.7)	1 (12.5)	0.673
*bla*_SHV_	19 (22.9)	18 (24.0)	1 (12.5)	0.675
*bla*_TEM_	8 (9.6)	8 (10.7)	0 (0.0)	>0.999
**AmpC**				
Screening of AmpC using cefoxitin (30 μg) resistance criteria	67 (80.7)	59 (78.7)	8 (100.0)	0.344
Phenotypic AmpC confirmation using cefoxitin (30 μg)/phenylboronic acid	10 (12.0)	10 (13.3)	0 (0.0)	0.587
Genotypic AmpC confirmation using PCR	21 (25.3)	21 (28.0)	0 (0.0)	0.193
*bla*_FOX_	5 (6.0)	5 (6.7)	0 (0.0)	>0.999
*bla*_ACT_	6 (7.2)	6 (8.0)	0 (0.0)	>0.999
*bla*_CMY_	4 (4.8)	4 (5.3)	0 (0.0)	>0.999
*bla*_LAT_	9 (10.8)	9 (12.0)	0 (0.0)	0.589
*bla*_DHA_	5 (6.0)	5 (6.7)	0 (0.0)	>0.999
**Carbapenemase and MBLs**				
Phenotypic carbapenemase confirmation (only mCIM positive)	28 (33.7)	26 (34.7)	2 (25.0)	>0.711
Genotypic carbapenemase confirmation using PCR	28 (33.7)	26 (34.7)	2 (25.0)	>0.711
*bla*_OXA–48–like_	15 (18.1)	15 (20.0)	0 (0.0)	0.34
*bla*_GES_	13 (15.7)	11 (14.7)	2 (25.0)	0.605
Phenotypic MBLs confirmation (both mCIM/eCIM positive)	20 (24.1)	18 (24.0)	2 (25.0)	>0.999
Genotypic MBLs confirmation using PCR	35 (42.2)	33 (44.0)	2 (25.0)	0.458
*bla*_NDM_	33 (39.8)	31 (41.3)	2 (25.0)	0.468
*bla*_IMP_	3 (3.6)	3 (4.0)	0 (0.0)	>0.999
*bla*_VIM_	6 (7.2)	6 (8.0)	0 (0.0)	>0.999

**Beta-lactamase genotypes and co-occurrence patterns**	**Total carbapenem-resistant *Klebsiella pneumoniae* isolates** ***n*: 83**	**CR-cKp** ***n*: 75**		**CR-hvKp** ***n*: 8**
	***n* (%)**	***n* (%)**		***n* (%)**

*bla*_CTX–M_	1 (1.2)	1 (1.3)		0 (0.0)
*bla*_LAT_	2 (2.4)	2 (2.7)		0 (0.0)
*bla*_DHA_	1 (1.2)	1 (1.3)		0 (0.0)
*bla*_ACT_	1 (1.2)	1 (1.3)		0 (0.0)
*bla*_GES_	5 (6.0)	3 (4.0)		2 (25.0)
*bla*_OXA–48–like_	4 (4.8)	4 (5.3)		0 (0.0)
*bla*_NDM_	11 (13.3)	9 (12.0)		2 (25.0)
*bla*_IMP_	2 (2.4)	2 (2.7)		0 (0.0)
*bla*_CTX–M_/*bla*_SHV_	6 (7.2)	5 (6.7)		1 (12.5)
*bla*_CMY_/*bla*_LAT_	1 (1.2)	1 (1.3)		0 (0.0)
*bla*_NDM_ */bla*_OXA–48–like_	1 (1.2)	1 (1.3)		0 (0.0)
*bla*_NDM_ */bla*_GES_	2 (2.4)	2 (2.7)		0 (0.0)
*bla*_NDM_ */bla*_FOX_	1 (1.2)	1 (1.3)		0 (0.0)
*bla*_NDM_ */bla*_VIM_	1 (1.2)	1 (1.3)		0 (0.0)
*bla*_NDM_ */bla*_DHA_	1 (1.2)	1 (1.3)		0 (0.0)
*bla*_NDM_ */bla*_VIM_ */bla*_GES_	1 (1.2)	1 (1.3)		0 (0.0)
*bla*_NDM_*/bla*_OXA–48–like_ */bla*_ACT_	1 (1.2)	1 (1.3)		0 (0.0)
*bla*_NDM_*/bla*_OXA–48–like_ */bla*_DHA_	1 (1.2)	1 (1.3)		0 (0.0)
*bla*_NDM_ */bla*_GES_ */bla*_LAT_	1 (1.2)	1 (1.3)		0 (0.0)
*bla*_GES_ */bla*_CMY_ */bla*_LAT_	1 (1.2)	1 (1.3)		0 (0.0)
*bla*_CTX–M_ */bla*_TEM_ */bla*_OXA–48–like_	1 (1.2)	1 (1.3)		0 (0.0)
*bla*_CTX–M_ */bla*_SHV_ */bla*_NDM_	2 (2.4)	2 (2.7)		0 (0.0)
*bla*_CTX–M_*/bla*_SHV_*/bla*_NDM_/*bla*_GES_	1 (1.2)	1 (1.3)		0 (0.0)
*bla*_CTX–M_*/bla*_SHV_*/bla*_NDM_/*bla*_OXA–48–like_	1 (1.2)	1 (1.3)		0 (0.0)
*bla*_CTX–M_ */bla*_SHV_ */bla*_TEM_ */bla*_OXA–48–like_	1 (1.2)	1 (1.3)		0 (0.0)
*bla*_CTX–M_*/bla*_SHV_*/bla*_OXA–48–like_ */bla*_DHA_	1 (1.2)	1 (1.3)		0 (0.0)
*bla*_CTX–M_ */bla*_SHV_ */bla*_TEM_ */bla*_ACT_	1 (1.2)	1 (1.3)		0 (0.0)
*bla*_CTX–M_ */bla*_SHV_ */bla*_TEM_ */bla*_DHA_	1 (1.2)	1 (1.3)		0 (0.0)
*bla*_CTX–M_ */bla*_SHV_ */bla*_TEM_ */bla*_NDM_	1 (1.2)	1 (1.3)		0 (0.0)
*bla*_CTX–M_ */bla*_SHV_ */bla*_TEM_ */bla*_GES_	1 (1.2)	1 (1.3)		0 (0.0)
*bla*_NDM_ */bla*_VIM_ */bla*_FOX_ */bla*_LAT_	1 (1.2)	1 (1.3)		0 (0.0)
*bla*_NDM_ */bla*_VIM_ */bla*_ACT_ */bla*_LAT_	1 (1.2)	1 (1.3)		0 (0.0)
*bla*_NDM_*/bla*_OXA–48–like_ */bla*_FOX_*/bla*_ACT_	2 (2.4)	2 (2.7)		0 (0.0)
*bla*_NDM_ */bla*_IMP_ */bla*_GES_ */bla*_CMY_ */bla*_LAT_	1 (1.2)	1 (1.3)		0 (0.0)
*bla*_CTX–M_ */bla*_SHV_ */bla*_TEM_ */bla*_FOX_ */bla*_CMY_	1 (1.2)	1 (1.3)		0 (0.0)
*bla*_CTX–M_ */bla*_SHV_ */bla*_NDM_ */bla*_VIM_ */bla*_OXA–48–like_	1 (1.2)	1 (1.3)		0 (0.0)
*bla*_CTX–M_ */bla*_SHV_ */bla*_TEM_ */bla*_NDM_ */bla*_VIM_ */bla*_OXA–48–like_	1 (1.2)	1 (1.3)		0 (0.0)

### Phenotypic and genotypic prevalence of AmpCs

Although using cefoxitin (30 μg) resistance criteria, 78.7% (*n* = 59/75) of CR-cKp and 100.0% (*n* = 8/8) of CR-hvKp were presumptive AmpC producers, the confirmatory cefoxitin (30 μg)/phenylboronic acid method was positive in only 13.3% (*n* = 10/75) and 0.0% (*n* = 0/8) of CR-cKp and CR-hvKp isolates, respectively (Table 5). However, the PCR showed that 28.0% (*n* = 21/75) of CR-cKp and none of CR-hvKp isolates were AmpC producers. The PCR method detected a greater number of AmpC positive CR-cKp isolates compared with the phenotypic confirmatory test. The distribution of AmpC genes among CR-cKp was as follows: *bla*_LAT_ (12.0%, *n* = 9/75), *bla*_ACT_ (8.0%, *n* = 6/75), *bla*_FOX_ (6.7%, *n* = 5/75), *bla*_DHA_ (6.7%, *n* = 5/75), and *bla*_CMY_ (5.3%, *n* = 4/75). The *bla*_ACC_ was not detected in any isolate (Table 5). The distribution of AmpCs was not significantly different in CR-cKp and CR-hvKp isolates (*P*-value > 0.05, Table 5).

### Phenotypic and genotypic prevalence of carbapenemases and metallo-beta-lactamases

Using phenotypic criteria (only mCIM positive), 34.7% (*n* = 26/75) and 25.0% (*n* = 2/8) of CR-cKp and CR-hvKp were carbapenemase producers, respectively (Table 5). PCR showed similar results compared with mCIM. All mCIM positive isolates carried at least one carbapenemase gene. The result of the mCIM test was consistent with that of PCR. The distribution of carbapenemase genes among CR-cKp was as follows: *bla*_OXA–48–like_ (20.0%, *n* = 15/75), *bla*_GES_ (14.7%, *n* = 11/75). Moreover, the occurrence of carbapenemase genes in CR-hvKp isolates was as follows: *bla*_OXA–48–like_ (0.0%, *n* = 0/8), *bla*_GES_ (25.0%, *n* = 2/8) (Table 5). The *bla*_KPC_ and *bla*_IMI_ were not detected in any isolate. The distribution of carbapenemases was not significantly different in CR-cKp and CR-hvKp isolates (*P*-value > 0.05, Table 5).

In phenotypic evaluation (both mCIM/eCIM positive), 24.0% (*n* = 18/75) and 25.0% (*n* = 2/8) of CR-cKp and CR-hvKp were MBL positive, respectively (Table 5). The PCR showed that all mCIM/eCIM positive isolates carried at least one MBL gene. However, the PCR method detected a greater number of MBL positive isolates compared with the phenotypic confirmatory criteria. Using PCR, 44.0% (*n* = 33/75) of CR-cKp and 25.0% (*n* = 2/8) of CR-hvKp isolates were MBL producers. The distribution of MBL genes among CR-cKp was as follows: *bla*_NDM_ (41.3%, *n* = 31/75), *bla*_VIM_ (8.0%, *n* = 6/75), and *bla*_IMP_ (4.0%, *n* = 3/75). Moreover, 25.0% (*n* = 2/8) of CR-hvKp isolates carried *bla*_NDM_ gene (Table 5). The *bla*_VIM_ and *bla*_IMP_ genes were not detected in any CR-hvKp isolate. The distribution of MBLs was not significantly different in CR-cKp and CR-hvKp isolates (*P*-value > 0.05, Table 5).

### Beta-lactamase genotypes

The different genotypes of beta-lactam resistance genes were shown in Table 5. According to Table 5, 37 and 3 different gene profiles were detected among the 75 CR-cKp and 8 CR-hvKp isolates, respectively. The *bla*_NDM_ gene was the most prevalent pattern, found in 12.0% (*n* = 9/75) of CR-cKp and 25.0% (*n* = 2/8) of CR-hvKp isolates. Also, the *bla*_CTX–M_/*bla*_SHV_ was the second most prevalent pattern, detected in 6.7% (*n* = 5/75) of CR-cKp and 12.5% (*n* = 1/8) of CR-hvKp isolates. The frequency of co-occurrence of various beta-lactamases was as follows: MBL/carbapenemase/AmpC (8.0%, *n* = 6/75), MBL/carbapenemase/ESBL (5.3%, *n* = 4/75), carbapenemase/ESBL/AmpC (1.3%, *n* = 1/75), MBL/carbapenemase and MBL/AmpC (each 5.3%, *n* = 4/75), ESBL/carbapenemase, ESBL/MBL, and ESBL/AmpC (each 4.0%, *n* = 3/75), and carbapenemase/AmpC (1.3%, *n* = 1/75).

### Molecular typing of CR-cKp and CR-hvKp isolates by enterobacterial repetitive intergenic consensus polymerase chain reaction

**FIGURE 1:**
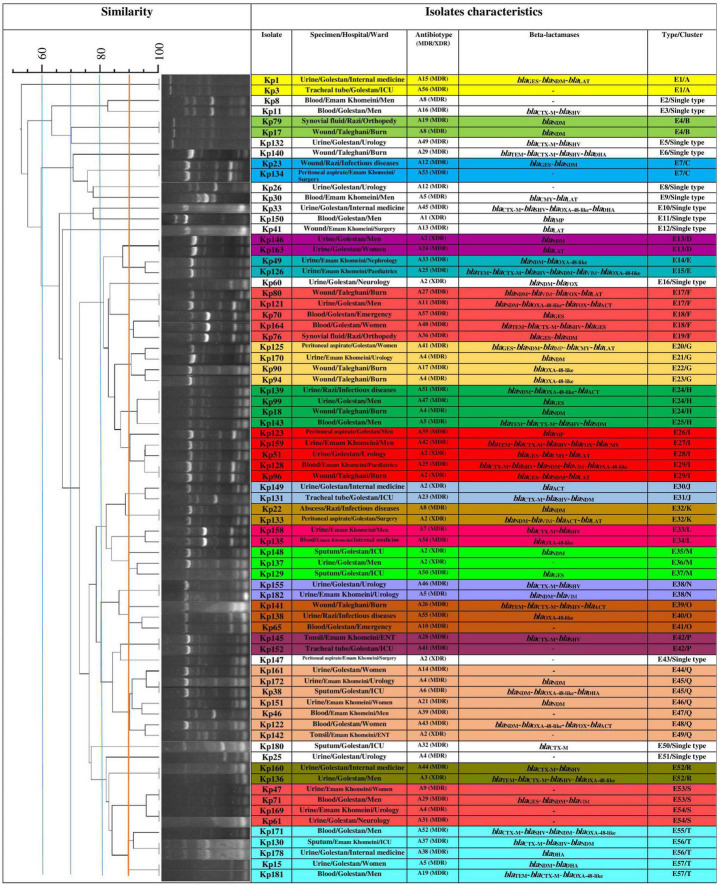
Clonal relatedness of 75 carbapenem-resistant classic *Klebsiella pneumoniae* using ERIC-PCR. Based on the UPGMA and Dice similarity coefficient, the isolates were assigned to 20 clusters and 13 singletons.

**FIGURE 2:**
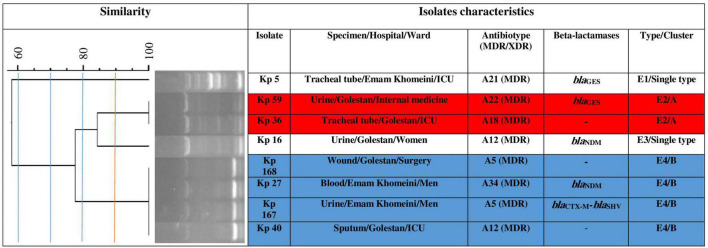
Clonal relatedness of eight carbapenem-resistant hypervirulent *Klebsiella pneumoniae* using ERIC-PCR. Based on the UPGMA and Dice similarity coefficient, the isolates were assigned to two clusters and three singletons.

Using ERIC-PCR at 90.0% similarity cut off value, the 75 CR-cKp isolates were assigned to 20 clusters (A to T) consisting of 2–7 isolates (82.7%, *n* = 62/75) and 13 singletons (17.3%, *n* = 13/75) with 57 different ERIC-types (E1–E57), indicating high genetic diversity among the isolates (Figure 1). Electrophoresis of PCR amplicons indicated 3–10 bands with various sizes ranged from 100 bp to about 1300 bp for each isolate. The cluster Q with seven isolates was the most predominant type, followed by F, I, T (each five isolates), G, H, S (each four isolates), M, O (each three isolates), A, B, C, D, E, J, K, L, N, P, and R (each two isolates) (Figure 1). The XDR isolates were seen in clusters D, I, J, K, M, Q, R and ERIC-types E11, E13, E16, E28, E29, E30, E32, E35, E36, E43, E49, and E52. The ERIC-type E4/cluster B isolates had the same beta-lactamase background (*bla*_NDM_ positive). The remaining isolates had different beta-lactamase genotypes. The eight CR-hvKp isolates were also divided into two clusters (A and B) and two singletons with four ERIC -types (E1–E4) (Figure 2). Electrophoresis of the PCR products showed 2–8 bands for each isolate with different sizes ranging from 100 bp to about 800 bp. Based on the data from ERIC-PCR, the CR-hvKp isolates were found to have lower genetic diversity compared to CR-cKp isolates. However, a wide range of antibiotypes and beta-lactamase genotypes was found among the CR-hvKp isolates, even among those with same cluster and ERIC-types.

## Discussion

In this study, based on phenotypic and molecular methods, 153 *K. pneumoniae* isolates, including 136 (88.9%) cKp and 17 (11.1%) hvKp isolates, were identified in different clinical samples. Previous reports from Iran by Davoudabadi et al. (2022) (29.9%, *n* = 14/52), Rastegar et al. (2019) (15.1%, *n* = 22/146), and Pajand et al. (2020) (2.5%, *n* = 3/122) showed different prevalence rates of hvKp than the current study. However, Örsten et al. (2020) (10.8%, *n* = 12/111) from Turkey reported almost the same prevalence of hvKp as in the current study. These discrepancies can be explained by the different nature and size of the samples studied and the detection method of the hvKp isolates. To date, there are no standard methods to differentiate hvKp from cKp strains, and several studies classified hvKp based on positivity of the string test and some virulence genes including *rmpA, rmpA2, iucA, peg-344*, and *magA* (Rastegar et al., 2019; Liao et al., 2020; Örsten et al., 2020; Serban et al., 2021; Zhu et al., 2021; Davoudabadi et al., 2022). In this study, 35.3% (*n* = 6/17) of hvKp had a positive string test, and all (100.0%, *n* = 6/6) were also positive for at least one of the studied genes. However, 64.7% (*n* = 11/17) of hvKp isolates carrying gene markers had a negative string test. This phenomenon has also been reported in previous studies from Turkey and Iran (Örsten et al., 2020; Davoudabadi et al., 2022). This may be due to the presence of mutations in the studied genes that prevent their function to produce the hypervirulent phenotype (Yang et al., 2022). The occurrence of different genes among the 17 hvKp isolates was as follows: *magA* (11.8%, *n* = 2), *rmpA* (11.8%, *n* = 2), *rmpA2* (52.9%, *n* = 9), *iucA* (52.9%, *n* = 9), and *peg344* (35.3%, *n* = 6). Simultaneous occurrence of two or more genes was also found in five (29.4%) isolates. In a previous study from Turkey, string test, *iucA*, and *magA* were positive in three (25.0%), eight (66.7%0, and one (8.0%) of the hvKp isolates, respectively. However, the *rmpA* and *peg344* genes were not detected (Örsten et al., 2020). Moreover, previous studies from China (Liu and Guo, 2018) and Iran (Rastegar et al., 2019; Davoudabadi et al., 2022) found different occurrence rates for hvKp-associated gene markers. These different results suggest that the phenomenon of hypervirulence is a complex process to which multiple genetic factors contribute, rather than a single gene (Hwang et al., 2022).

In this study, the results of antibiotic susceptibility testing (AST) revealed that a relatively high proportion (more than 50.0%) of cKp and hvKp isolates were resistant to aminoglycosides, beta-lactam combination agents (piperacillin-tazobactam and ampicillin/sulbactam), quinolones, folate pathway inhibitors, and different classes of cephalosporins. These findings were consistent with recent reports of increasing emergence of highly resistant XDR and PDR cKp and hvKp strains from different countries, including Iran, Turkey, Spain, India, Saudi Arabia, and Lebanon (Örsten et al., 2020; Ballén et al., 2021; Banerjee et al., 2021; Sleiman et al., 2021; Badger-Emeka and Emeka, 2022; Davoudabadi et al., 2022). Generally, this can be explained by the presence of mobile genetic elements (MGEs) harboring several antibiotic resistance genes including IncFI, ColKP3, and IncR plasmids in the cKp and hvKp isolates (Xanthopoulou et al., 2020; Banerjee et al., 2021). Also, 55.1% (*n* = 75/136) of cKp and 47.1% (*n* = 8/17) of hvKp isolates were CR-cKp and CR-hvKp, respectively and showed various resistance rates against ertapenem, imipenem, and meropenem ranging from 47.1 to 54.4% (Table 2). However, 80.0% of CR-cKp and all CR-hvKp isolates were simultaneously resistant to ertapenem, imipenem, and meropenem. Previous studies from Iran by Rastegar et al. (2019) and Davoudabadi et al. (2022) reported lower resistance rates to carbapenems in cKp and hvKp isolates (ranging from 9.1 to 42.5%). There are a number of reasons that could explain these inconsistencies, including the source of isolates, sample type and size, different patient populations and races, and treatment regimens commonly prescribed in the region.

Another finding of the current study was the high susceptibility of cKp, hvKp, CR-cKp, and CR-hvKp isolates to colistin and tigecycline. These two antibiotics, together with tetracycline, were the most effective drugs against studied isolates, so that all CR-cKp isolates were susceptible to them. Moreover, only 4.0% (*n* = 3/75) of CR-cKp isolates were resistant to colistin. These observations coincided well with previous reports from Iran (Maleki et al., 2018; Davoudabadi et al., 2022), Spain (Ballén et al., 2021), and China (Shao et al., 2021; Yang et al., 2022). Perhaps these results were not entirely unexpected, as these drugs are not usually part of standard treatment regimens in our region and are used only as a last resort and for infections caused by MDR bacteria due to the severe side effects of antibiotics such as colistin (Chen et al., 2021; Moghadam et al., 2022). Nevertheless, the increasing emergence of colistin and tigecycline resistant *Klebsiella* strains, especially hvKp, poses a serious challenge to healthcare systems (Chen et al., 2021; Liu et al., 2022). Resistance to colistin in carbapenem-resistant *K. pneumoniae* has been reported as high as 50.0% in Iran (Jafari et al., 2019).

In this study, the majority of CR-cKp and CR-hvKp isolates had high resistance rates (more than 60.0%) against 18 of 23 tested antibiotics, so that 84.0% (*n* = 63/75) and 16.0% (*n* = 12/75) of CR-cKp were MDR and XDR, respectively. While all CR-hvKp (100.0%, *n* = 8) isolates were MDR (Table 2). Also, the majority of the carbapenem-resistant isolates (84.3%, *n* = 70/83) had a MARI of ≥0.5 and no PDR isolate was detected. In contrast to the current study, in previous reports by Örsten et al. (2020) from Turkey, Davoudabadi et al. (2022) from Iran, and Banerjee et al. (2021) from India, XDR and PDR CR-hvKp isolates have been reported. The lack of integrated stewardship programs for antibiotic use and prescribing and the sale of antibiotics in pharmacies without a doctor’s prescription are among the reasons for the increasing antibiotic resistance rates in GNB, especially in third world countries (Sharma et al., 2022).

In contrast to previous studies (Liu and Guo, 2018; Rastegar et al., 2019; Sanikhani et al., 2021), in which the antibiotic resistance rates of hvKp isolates to several antibiotics were significantly lower than those of cKp strains, no significant difference was found between the cKp with hvKp and CR-cKP with CR-hvKp isolates in this study, except for tigecycline. These findings was in line with the previous study by Taraghian et al. (2020) from Iran, where no significant difference was found. It is likely that the acquisition of genetic factors carrying resistance genes by hvKp has increased these resistance rates over time (Sanikhani et al., 2021).

In this research, most CR-cKp (42.7%, *n* = 32/75) and CR-hvKp (37.5%, *n* = 3/8) isolates were identified from urine samples, followed by blood samples for CR-cKp (20.0%, *n* = 15/75) and tracheal tubes (25.0%, *n* = 2/8) for CR-hvKp. Also, the CR-hvKp isolates were more prevalent in the ICU ward (37.5%, *n* = 3/8) than in other wards. However, the distribution of CR-cKp and CR-hvKp did not differ significantly by sample type, hospital wards, and other demographic variables (*P* > 0.05) (Table 4). These findings were consistent with previous observations by Rastegar et al. (2019) and Sanikhani et al. (2021) from Iran, who found the highest prevalence of hvKp isolates in the urine and respiratory samples and in ICU ward. Similar to our results, they also found no significant difference between the occurrence of cKp and hvKp in terms of sample type, hospital wards, and other demographic variables (Rastegar et al., 2019; Sanikhani et al., 2021). However, the frequency of hvKp in the men ward was significantly higher than that of cKp (*P*-value = 0.049) in this study. The reason for this observation was unclear.

One of the strengths of the current study was the investigation of a wide variety of beta-lactamases, including AmpC enzymes in CR-hvKp isolates, which were not addressed in previous studies from Iran. This study elucidated the prevalence of ESBLs, carbapenemases, MBLs, and AmpCs among CR-cKp and CR-hvKp isolates in southwestern Iran. Using CDT test, 26.7% (*n* = 20/75) and 12.5% (*n* = 1/8) of CR-cKp and CR-hvKp were ESBL producers, respectively. The PCR assay confirmed the results of the CDT phenotypic test and all CDT positive isolates harbored at least one ESBL gene. Also, the co-occurrence of ESBL genes was detected in both CR-cKp and CR-hvKp isolates, which was in line with previous reports from different countries (Hamzaoui et al., 2018; Taraghian et al., 2020; Banerjee et al., 2021; Sanikhani et al., 2021). The following occurrences were observed for the ESBL genes among CR-cKp: *bla*_CTX–M_ (26.7%, *n* = 20/75), *bla*_SHV_ (24.0%, *n* = 18/75), *bla*_TEM_ (10.7%, *n* = 8/75); and for CR-hvKp: *bla*_CTX–M_ (12.5%, *n* = 1/8), *bla*_SHV_ (12.5%, *n* = 1/8), and *bla*_TEM_ (0.0%, *n* = 0/8) (Table 5). None of the isolates were positive for *bla*_PER_ and *bla*_VEB_ genes. Moreover, the distribution of ESBLs was not significantly different in CR-cKp and CR-hvKp isolates (*P*-value > 0.05, Table 5). A previous report from Iran by Davoudabadi et al. (2022) showed a different occurrence rate of ESBL genes in hvKp isolates as follows: *bla*_CTX–M_ (21.4%, *n* = 3/14), *bla*_SHV_ (28.6%, *n* = 4/14), and *bla*_TEM_ (78.6%, *n* = 11/14). However, similar to the findings of the current study, the *bla*_PER_ and *bla*_VEB_ were not detected (Davoudabadi et al., 2022). Also, Taraghian et al. (2020) from Iran reported the following occurrence rates of ESBLs in cKp and hvKP, respectively: *bla*_SHV_ (90.9, 60.6%), *bla*_TEM_ (63.6, 58.5%), and *bla*_CTX–M_ (63.6, 57.4%). These frequencies were higher than in the current study. Moreover, in contrast to this research, the prevalence of *bla*_SHV_ was significantly higher (*P* = 0.048) in hvKp isolates than in cKp strains (Taraghian et al., 2020). In another study by Banerjee et al. (2021) from India, all CR-hvKp (*n* = 9) harbored *bla*_SHV_ and *bla*_CTX–M_, whereas *bla*_TEM_ was not detected. In this study, the *bla*_CTX–M_ was the most frequent ESBL among carbapenem-resistant *K. pneumoniae* isolates that was in line with the previous report from Iraq (Raouf et al., 2022). In the last decade, *bla*_TEM_ and *bla*_SHV_ variants have become less dominant than *bla*_CTX–M_, despite being the most universal ESBLs (Soltani et al., 2020; Raouf et al., 2022). Antibiotic use plans differ in each region, which may cause selection pressure for the circulation of ESBL-producing isolates and explain these inconsistencies. Also, the origin of collected samples and the geographical variances may contributed to these differences (Raouf et al., 2022).

In this study, AmpC genes were not detected in CR-hvKp isolates. However, several AmpC genes were detected in 28.0% (*n* = 21/75) of CR-cKp isolates as follows: *bla*_LAT_ (12.0%, *n* = 9/75), *bla*_ACT_ (8.0%, *n* = 6/75), *bla*_FOX_ (6.7%, *n* = 5/75), *bla*_DHA_ (6.7%, *n* = 5/75), *bla*_CMY_ (5.3%, *n* = 4/75), and *bla*_ACC_ (0.0%, *n* = 0/75). There is evidence for the role of AmpC beta-lactamase in resistance to carbapenems and increasing their MIC (Kazemian et al., 2019). In a previous observation from Iran, higher prevalence rates of *bla*_CMY–2_ (60.7%) and *bla*_DHA–1_ (34.4%) were found in *K. pneumoniae* isolates (Soltani et al., 2020). To the best of our knowledge, *bla*_DHA_ AmpC harboring hvKp ST23 have been sporadically reported from China (Xie et al., 2018; Xu et al., 2018) and Korea (Cheong et al., 2016). So far, no reports have clarified the presence of AmpCs in hvKp isolates from Iran. There were differences between the results of AmpC confirmatory phenotypic testing and PCR in the present study, so that the PCR method detected more number of AmpC positive CR-cKp isolates. This observation was similar to previous reports from Iran (Kazemian et al., 2019), which showed a high rate of false-negative results by AmpC phenotypic test.

In this study, 34.7% (*n* = 26/75) of CR-cKp and 25.0% (*n* = 2/8) of CR-hvKp, were carbapenemase producers using mCIM test. All mCIM positive isolates carried at least one carbapenemase gene. The distribution of carbapenemase genes among CR-cKp and CR-hvKp was as follows: *bla*_OXA–48–like_ (20.0, 0.0%) and *bla*_GES_ (14.7, 25.0%). Also, the *bla*_KPC_ and *bla*_IMI_ were not detected in any isolate. This study was the first to detect the *bla*_GES_ carbapenemase in CR-hvKp isolates from Iran. In previous studies from Iran, *bla*_GES_ was not detected in hvKp isolates (Nasiri et al., 2020; Sanikhani et al., 2021; Davoudabadi et al., 2022). However, according to a systematic review and meta-analysis from Iran, the *bla*_GES_ gene (27.8%) was the third most prevalent carbapenemase among carbapenem-resistant *K. pneumoniae* isolates (Nasiri et al., 2020). The presence of *bla*_GES_ gene in CR-hvKp isolates has been reported in wastewater samples and nasal swabs that were obtained from the employees of wastewater treatment plants from Poland (Rolbiecki et al., 2021). Also, similar to previous investigations from Iran, no hvKp isolate harbored *bla*_KPC_ carbapenemase in the current study (Tabrizi et al., 2018; Sanikhani et al., 2021; Davoudabadi et al., 2022). China has the highest prevalence of *bla*_KPC_ carbapenemase, and 80.0% of circulating CR-hvKp strains belong to ST11 clone that carry KPC-2 (Lan et al., 2021). This carbapenemase has also been reported from Singapore, USA, Canada, and Argentina (Lan et al., 2021). Previous reports by Sanikhani et al. (2021) and Davoudabadi et al. (2022) from Iran, have reported the occurrence of 14.3 and 53.9% for *bla*_OXA–48_ in hvKp isolates, respectively. In a systematic review and meta-analysis by Nasiri et al. (2020) from Iran, the *bla*_OXA–48_ gene (47.1%) was reported to be the most prevalent gene in carbapenem-resistant *K. pneumoniae* isolates.

In this study, 24.0% (*n* = 18/75) of CR-cKp and 25.0% (*n* = 2/8) of CR-hvKp isolates were MBL positive using the mCIM/eCIM positivity criteria. PCR showed that all mCIM/eCIM positive isolates carried at least one MBL gene. However, PCR detected a greater number of MBL positive isolates than the phenotypic confirmatory test. This may be due to the co-occurrence of carbapenemases and MBLs, resulting in a false-negative eCIM test (Clinical and Laboratory Standards Institute, 2021). Using PCR, 44.0% (*n* = 33/75) of CR-cKp and 25.0% (*n* = 2/8) of CR-hvKp isolates were MBL producers. The *bla*_NDM_ gene was the most frequently detected MBL gene in CR-cKp (41.3%) and CR-hvKp isolates (25.0%), followed by *bla*_VIM_ (8.0%) and *bla*_IMP_ (4.0%) for CR-cKp. These results were in good parallel with available data that identified the *bla*_NDM_ (30.1%) as the most predominant MBL type among *K. pneumoniae* isolates from Iran, followed by *bla*_VIM_ (10.6%) and *bla*_IMP_ (4.5%) (Nasiri et al., 2020). However, the *bla*_VIM_ and *bla*_IMP_ genes were not detected in CR-hvKp isolates, which was in contrast to a previous study by Tabrizi et al. (2018) from Iran that showed the emergence of CR-hvKp isolates carrying *bla*_VIM–2_. Also, in line with the current study, *bla*_VIM_ and *bla*_IMP_ genes were not detected by Davoudabadi et al. (2022) in hvKp isolates from Iran. Meanwhile, they found the *bla*_NDM_ in 7.1% (*n* = 1/14) of hvKp isolates (Davoudabadi et al., 2022). In most Asian countries, *bla*_NDM_ and *bla*_OXA–48_ genes were frequently associated with hvKp isolates (Banerjee et al., 2021). This phenomenon could be the consequence of antibiotic selection pressure leading to the hvKP isolates acquiring plasmids with multiple antibiotic resistance genes (carbapenemase, ESBLs, colistin resistance genes) (Banerjee et al., 2021). Another finding of the current study was the co-existence of different carbapenemases, ESBLs, AmpCs, and MBLs in CR-cKp isolates. MBL/carbapenemase/AmpC (8.0%, *n* = 6/75) was the most co-occurrence pattern among CR-cKp isolates. In contrast, CR-hvKp isolates had only the ESBL co-existence pattern (*bla*_CTX–M_/*bla*_SHV)_. Contrary to these results, the co-occurrence of ESBLs and carbapenemases in hvKp isolates has been reported in previous studies from Iran (Sanikhani et al., 2021; Davoudabadi et al., 2022).

In this study, all carbapenem-resistant isolates were typeable by ERIC-PCR. ERIC-PCR showed higher genetic diversity among CR-cKp compared with CR-hvKp isolates. Accordingly, the CR-cKp isolates were assigned to 20 clusters and 13 singletons, while the CR-hvKp isolates were categorized into two clusters and three singletons. These results suggest that the spread and circulation of the CR-hvKp strains was due to the occurrence of dependent clones. However, the CR-cKp and CR-hvKp isolates with similar ERIC-types had a wide range of antibiotypes and beta-lactamase genotypes. This diversity could complicate the treatment of infections in our region. These results were in accordance with previous reports by Sedighi et al. (2020) from Hamadan, Iran and Abdelhamid et al. (2020) from Egypt. For genotyping of bacteria including *K. pneumoniae* isolates, several expensive and time-consuming molecular methods such as pulsed-field gel electrophoresis (PFGE), multiple locus sequence typing (MLST), and ribotyping are available (Mohammadi Bardbari et al., 2020; Sedighi et al., 2020). However, ERIC-PCR method is an inexpensive, reliable, and fast molecular method for genotyping of *Enterobacteriaceae* family (Sedighi et al., 2020).

This study had some limitations, including the lack of capsular type determination of hvKp isolates. Also, due to the traffic restrictions caused by the COVID-19 pandemic and the lack of services by many companies and enough financial sources, the sequence of all beta-lactamases was not determined which was another limitation of the current research.

## Future perspectives

The whole genome sequencing is recommended to elucidate a very important piece of in-depth information such as single nucleotide polymorphisms (SNPs) based analysis for phylogenetic relatedness, pan-genome analysis and insights on the circulating plasmids and Inc groups especially among the colistin-resistant isolates.

## Conclusion

This study was the first report of emergence of MDR CR-hvKp isolates harboring different beta-lactamases, including *bla*_CTX–M_, *bla*_SHV_, *bla*_NDM_, and *bla*_GES_, in southwestern Iran. As a result of their hypervirulence coupled with multidrug resistance, these isolates pose a particular threat to healthcare systems. Hence, long-term surveillance and more effective treatment strategies should be implemented to prevent the spread of CR-hvKp and reduce selection pressure. Also, it is recommended to use a fast and cheap molecular method such as ERIC-PCR for primary evaluation of clonal relatedness of MDR isolates.

## Data availability statement

The raw data supporting the conclusions of this article will be made available by the authors, without undue reservation.

## Ethics statement

This study was approved by the Ethics Committee of the Ahvaz Jundishapur University of Medical Sciences, Ahvaz, Iran (ethics code: IR.AJUMS.REC.1398.489) according to the Declaration of Helsinki. All methods were performed in accordance with the relevant guidelines and regulations of the Ethics Committee of the Ahvaz Jundishapur University of Medical Sciences, Ahvaz, Iran. Clinical samples were not collected as part of this study. The clinical samples were collected as routine clinical care and to check the presence of any infection for referred and admitted patients. As a result, written informed consent was waived by the Ethics Committee of Ahvaz Jundishapur University of Medical Sciences, Ahvaz, Iran.

## Author contributions

MSak performed the experiments, analyzed majority of the data, and wrote the whole manuscript. MSav, MH, and MA prepared all the necessary materials and performed some of the experiments. MSav, MH, and SSS contributed to the discussion of experimental results. MA designed and supervised the experiments at different stages. All authors contributed to the article and approved the submitted version.
